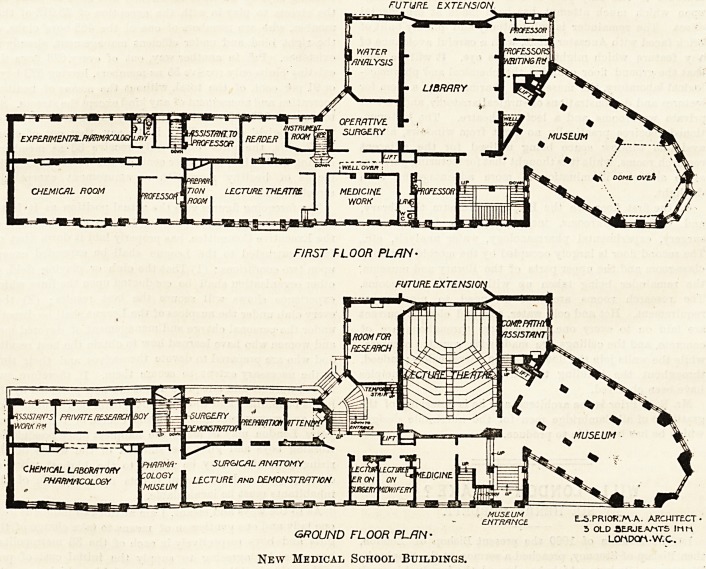# The New Science Buildings at Cambridge

**Published:** 1904-03-05

**Authors:** 


### HOSPITAL ADMINISTRATION.

### CONSTRUCTION AND ECONOMICS.

### THE NEW SCIENCE BUILDINGS AT CAMBRIDGE.

#### OPENING BY THE KING.

On Tuesday the King visited Cambridge in order to open the new buildings in Downing Street, in which will be housed the Schools of Geology, Botany, Law, and Medicine. His Majesty was joined at the railway station by the Queen nd Princess Victoria, who had travelled from Sandringham.

"The cordial welcome given to us by you on behalf of the County of Cambridge is very gratifying both to myself and to Queen Alexandra. I shall never forget my residence in your county while I was a student in the University which adds so greatly to its distinction. I am very glad to

The Humphry Museum.

Addresses of welcome were presented to the King by the Mayor and Corporation and the County Council. His Majesty, having warmly thanked the Corporation, went on to say:—

know of the educational work in connection with the great industry of agriculture which you have undertaken. In common with most branches of industry, agriculture has in modern times come to depend for its success and extension upon the unremitting application to it of the results of scientific investigation. No greater service can be rendered to this ancient industry than to furnish it with the means of research and instruction which are essential in order that labour may be directed in profitable channels. In the buildings which are to be opened to-day important provision will be made for instruction in some of the branches of knowledge upon which the scientific cultivation of the land is based, and you will, I hope, derive therefrom useful aid in the carrying out of your undertaking."

In the Senate House, the Presidents of the Royal Colleges of Physicians and Surgeons were conspicuous among the vast company who enthusiastically greeted the King and Queen on their arrival. The Vice-Chancellor having read the address of Chancellor, masters, and scholars of the University of Cambridge, his Majesty replied in the following terms:—

"I receive with much gratification your renewed expression of loyalty to my throne and person, and your sympathetic reference to my beloved mother, Queen Victoria, and my revered father, who was deeply interested in everything connected with the University of Cambridge, of which he was Chancellor. I am proud to be a member of the University, and I shall always look back with great pleasure upon the time which I spent as an undergrad of Trinity. It was a great satisfaction to me to send my dear son the Duke of Clarence to matriculate in the same ancient and splendid foundation, and I well remember with what pride and interest we saw, so shortly after our marriage, the University in which I studied. There are around us many monuments of the interest taken in the University by my ancestors; and, like them, I earnestly desire its well-being and the extension and development of all branches of study and research which are essential to the maintenance and the greatness and the welfare of my Empire. I have no doubt that Cambridge will continue to occupy a foremost place in this work. To the older Universities must succeed new endowments for education if my realm is to be kept up to its proper standard of efficiency. I am glad to know many munificent donations have been received to that end, and that the museums which are now being opened for the study of subjects of especial importance at the present time will serve as an example of that generosity, at which friends and supporters of the University will rejoice. I heartily thank you for your welcome to Queen Alexandra and myself, and I earnestly join with you in the prayer that my Empire may continue throughout my reign in peace and prosperity."

NEW MEDICAL SCHOOL BUILDINGS.

After luncheon in the Fitzwilliam Museum, the Royal party drove to the new buildings, which the King formally opened, also unveiling a statue of the late Professor Sedgwick.

### THE NEW MEDICAL SCHOOL BUILDINGS.

These buildings will provide accommodation for the departments of medicine, surgery, midwifery, and pharmacology, and for the present will also house the departments of public health, medical jurisprudence, and pathology. When the whole of the projected block is completed, and an Institute of Hygiene has been established, the last-mentioned three branches of medicine will be removed from the present buildings, thus allowing ample room for the purposes for which they will be required.

The block, as will be seen from the plans, consists of two wings meeting at a right angle, thus forming the shape of an ▟ . The long arm, which has a frontage of 100 feet on Downing Street, runs east and west. The short arm runs, from the eastern end of the long arm, northwards, and when finished will join the old buildings of the school. Projecting from the east face is the Humphry Museum, named after the late Sir George Humphry, and the cost of which was in large part defrayed by subscriptions from Cambridge medical graduates, many of whom had been pupils of the great Cambridge teacher and surgeon. The museum is the only part of the new medical buildings upon which much attempt has been made at ornamental effect. The remainder is substantially and plainly built of brick faced with Ancaster stone, with a careful avoidance of any feature which might offend the eye. It will be seen that the ground floor contains the chemical and pharmacological laboratory, the museum of pharmacology, a room for lectures and demonstrations of surgical anatomy, and various private workrooms and a lecture theatre. The last-mentioned receives practically no light from windows, all the available window space being utilised for the adjacent research rooms, while it is thought that for lecturing purposes alone artificial illumination is more advantageous than daylight.

On the first floor are the Humphry Museum, the library, and various workrooms, including those for operative surgery, experimental pharmacology, water analysis, etc., The second floor is largely occupied by the morbid histology class-room and the upper parts of the library and museum, the remainder being taken up with various workrooms. The research rooms are well arranged to meet every requirement. Hot and cold water, gas, and electric current are laid on to every one. The floors throughout are of concrete, and the ceilings are made with rounded cornices, while the walls join the floors in plain curves below; indeed, throughout the building the strictest hygienic principles have been observed.

Mr. E. S. Prior is the architect, and he is deserving of the gratitude of all Cambridge men for the admirable designs which he has been able to produce.

## Figures and Tables

**Figure f1:**
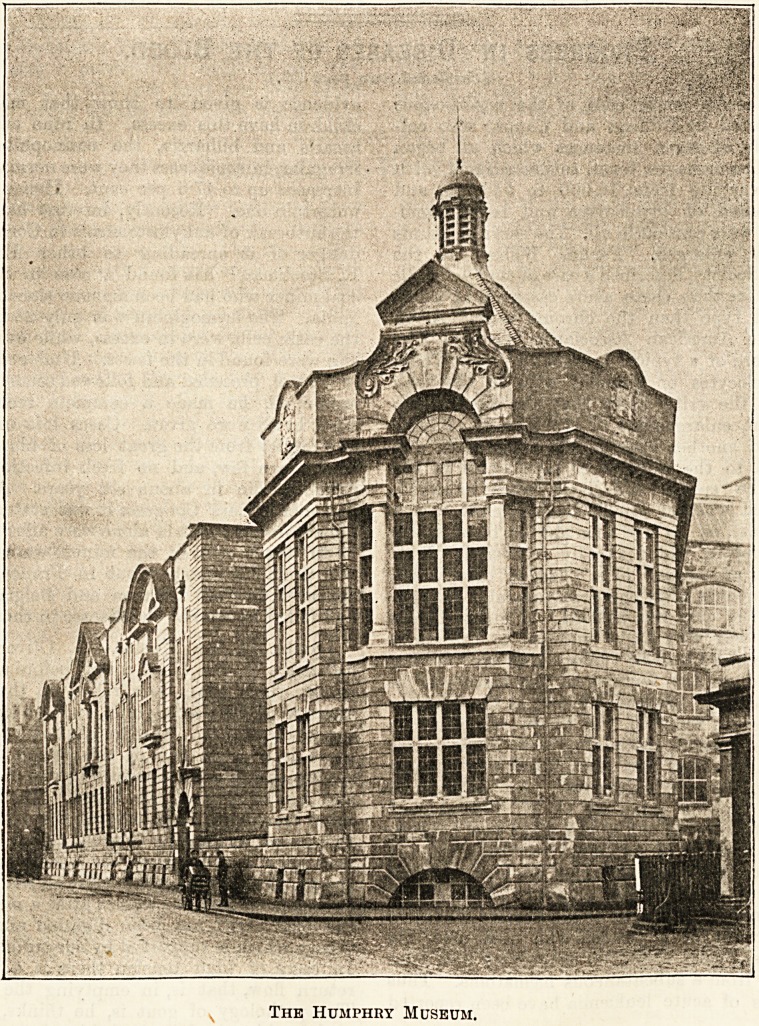


**Figure f2:**